# Effect of n-3 and n-6 unsaturated fatty acids on prostate cancer (PC-3) and prostate epithelial (RWPE-1) cells *in vitro*

**DOI:** 10.1186/1476-511X-12-160

**Published:** 2013-10-29

**Authors:** Hongzhou Meng, Yuzhen Shen, Junhui Shen, Feng Zhou, Shengrong Shen, Undurti N Das

**Affiliations:** 1 Department of Urology, The First Affiliated Hospital, Zhejiang University, Hangzhou 310003, China; 2 Department of Food Science and Nutrition, School of Biosystems Engineering & Food Science, Zhejiang University, Hangzhou 310058, China; 3 School of Medicine, Tongji University, Shanghai 200092, China; 4 UND Life Sciences, 2020 S 360th St, K-202, Federal Way, WA 98003, USA; 5 School of Biotechnology, Jawaharlal Nehru Technological University, Kakinada 533 003, India; 6 Department of Medicine, GVP Hospital and Bio-Science Research Centre, Campus of GVP College of Engineering, Visakhapatnam 530 048, India

**Keywords:** n-6 Polyunsaturated fatty acids, n-3 Polyunsaturated fatty acids, Prostate cancer, Cytokines, Free radicals, Lipid peroxidation, Lipoxin A4

## Abstract

Prostate cancer (PCa) is one of the leading causes of death in the elderly men. Polyunsaturated fatty acids (PUFAs) regulate proliferation of cancer cells. In the present study, we evaluated the effect of various PUFAs on the proliferation and survival of human prostate cancer (PC-3) and human prostate epithelial (RWPE-1) cells in vitro.

LA, GLA, AA, ALA, EPA and DHA (linoleic acid, gamma-linolenic acid, arachidonic acid, alpha-linolenic acid, eicosapentaenoic acid and docosahexaenoic acid respectively) when tested at 50, 100, 150, and 200 μM inhibited proliferation of RWPE-1 and PC-3 cells, except that lower concentrations of LA (25 μM) and GLA (5, 10 μM) promoted proliferation. Though all fatty acids tested produced changes in the production of interleukin-6 (IL-6), tumor necrosis factor-α (TNF-α), lipoxin A_4_ and free radical generation by RWPE-1 and PC-3 cells, there were significant differences in their ability to do so. As expected, supplementation of various n-3 and n-6 fatty acids to RWPE-1 and PC-3 cells enhanced the content of the added fatty acids and their long-chain metabolites in these cells. In contrast to previous results, we did not find any direct correlation between inhibition of cell proliferation induced by various fatty acids and free radical generation. These results suggest that polyunsaturated fatty acids suppress proliferation of normal and tumor cells by a variety of mechanisms that may partly depend on the type(s) of cell(s) being tested and the way these fatty acids are handled by the cells. Hence, it is suggested that more deeper and comprehensive studies are needed to understand the actions of fatty acids on the growth of normal and tumor cells.

## Introduction

Prostate cancer is one of the leading causes of cancer death among men in the United States [1]. Prostate cancer mortality rates are especially high in Northern Europe and Northern America and much lower in Asian countries [2,3]. Furthermore, immigrates from Asian countries have a significant increase in the risk of developing prostate cancer after residence in America [4], which suggests that, perhaps, western diets contribute to the development and progression of prostate cancer [5].

Compared to Eastern diet, Western diet contains higher levels of saturated fats, which may play a role in prostate cancer development [6]. The role of dietary fats on the risk of prostate cancer is controversial [7]. South American studies showed that only α-linolenic acid influenced the progression of prostate cancer. Several studies reported that n-3 PUFAs have an inverse association with prostate cancer, which showed that EPA and DHA suppressed human prostate tumor cell growth. This could be related to the fact that EPA and DHA can displace AA from cell membrane phospholipids and suppress pro-inflammatory prostaglandin synthesis [8]. However, findings from other studies have been inconsistent, some of which found positive association but others found weak association between unsaturated fatty acid intake and prostate cancer. In view of these conflicting results, we performed a detailed study on the effect of various unsaturated fatty acids on the growth of prostate cancer cells *in vitro* and the mechanisms involved therein.

Experimental studies showed that n-3 PUFAs such as EPA and DHA that are derived from essential fatty acid (EFA) ALA can suppress the growth of prostate cancer cells, whereas n-6 PUFAs such as GLA and AA, which are derived from EFA LA promote prostate tumor carcinogenesis [9,10]. Epidemiological studies were inconclusive on the association between prostate cancer risk and intake of n-3 or n-6 PUFAs that are substrates for eicosanoid synthesis, with n-6 PUFAs being converted predominantly into pro-inflammatory eicosanoids, while n-3 PUFAs being converted into anti-inflammatory or less pro-inflammatory eicosanoids [11]. There is increasing evidence that lipid metabolism plays an important role in various cellular processes, including cell proliferation, apoptosis, motility and inflammatory responses that may contribute to different aspects of tumorigenesis [12,13].

Tumor necrosis factor (TNF)-α can behave both as a pro-apoptotic factor and also a survival factor of tumor cells. Tumor cells themselves and the surrounding normal cells have the ability to secrete TNF-α that is a strong promoter of interleukin (IL)-6, another cytokine, that influences ERK/MAPK signaling pathway and thus, may show anti-proliferative and pro-apoptotic effect in PC-3 cell [14]. IL-6 is a pleiotropic cytokine regulating gene expression and interacting with B-cell and T-cell, and secreted by both hormone-dependent (LNCaP) and hormone-refractory (DU-145, PC-3) cells [15]. IL-6 may induce the development of melanoma, renal cell carcinoma, ovarian carcinoma, multiple myeloma, prostate carcinoma and breast cancer [16]. This implies that resolution of inflammation may lead to regression of cancer. In this context, it is noteworthy that AA, EPA and DHA form precursors to anti-inflammatory and pro-resolution modulators such as lipoxins, resolvins and protectins that may contribute to resolution of inflammation and thus, aid in the regression of tumor [17,18].

In addition, free radicals generated during cellular respiration and normal metabolism also have a modulatory influence on tumor cell growth. Free radicals when generated in excess damage DNA that, in turn, may initiate the development of cancer. PUFAs being highly unsaturated are easily targeted by free radicals to generate lipid peroxides that are toxic to cells. Both free radicals and lipid peroxides may serve as double edged sword: when produced in normal cells damage DNA and initiate the carcinogenic process; but when produced in sufficient amounts in tumor cells may actually kill tumor cells in view of their cytotoxic properties [19-22]. Thus, the role of PUFAs and their metabolites in cancer may depend on the way they are handled by normal and tumor cells.

In order to understand the role of various PUFAs on the growth of normal and tumor cells, we studied the effect of various PUFAs: LA, GLA, AA, ALA, EPA and DHA on the growth of RWPE-1(human prostate epithelial cell) and PC-3 (human prostatic carcinoma cell) and their effects on fatty acid metabolism and ability to modulate the production of IL-6, TNF-α, lipoxin A_4_ and free radical generation and the results are reported here.

## Materials and methods

### Materials

ALA, LA, GLA, AA, EPA and DHA were purchased from Sigma (USA). 20 mmol/l stock solutions of both ALA and LA were prepared in 0.1 mol/l NaOH respectively, and then further diluted in sterile water until the concentrations to be used in the experiments was obtained, whereas 20 mmol/l stock solutions of GLA, AA, EPA, DHA were prepared in anhydrous ethanol and further diluted in RPMI 1640 medium (GIBCO) until the concentrations needed to be used in the experiments was reached.

### Cell culture

Human prostatic carcinoma (PC-3) cell line and human prostate epithelial (RWPE-1) cell line were purchased from Institute of Biochemistry and Cell Biology, Chinese Academy of Sciences (Shanghai, China). Cells were grown in RPMI 1640 medium (GIBCO) supplemented with 100 U/ml penicillin, 100 U/ml streptomycin and 10% fetal bovine serum and grown 37°C in humidified air containing 5% CO_2_. The culture medium was replaced every 3 days.

### Cell proliferation assays

MTT(3-[4,5-dimethythiazol-2-yl]-2,5- diphenyltetrazolium bromide) assay was used for the determination of the number of viable cells in cell proliferation assays [23]. Both PC-3 and RWPE-1 cells were plated in 96-well plates with a volume of 190 μl at a density of 5 × 104 cells/ml. The cells were incubated with different concentrations of LA, GLA, AA, ALA, EPA and DHA for 24, 48, 72 h. At the end of incubation period, medium was removed and 20 μL of 5 mg/ml MTT was added to each well, incubated for 4 hours at 37°C and optical densities were read by a microplate reader at 490 nm.

### Lipoxin A_4_ measurement

The production of LXA_4_ in cell culture supernatant was measured by ELISA kit [24]. Both PC-3 cells and RWPE-1 cells were inoculated in 6-well plates in a volume of 3 ml at a density of 1 × 10^5^ cells/ml and incubated for 24 h. The supernatant of the cells (PC-3 and RWPE-1) that were treated with different concentrations of various fatty acids (LA: 50, 150 μM; GLA: 25, 50, 75 μM; AA:50, 100 μM; ALA:50, 150 μM; EPA:50, 100 μM and DHA: 50, 100 μM) for 48 h was collected for the measurement of lipoxin A4 and was quantified using LXA_4_ ELISA kit according to the manufacturer’s specifications (Source Leaf Biological Technology, Shanghai, China).

### IL-6 and TNF-α production

Cells were seeded in 6-well plates in a volume of 3 ml at a density of 1 × 10^5^ cells/ml (PC-3 and RWPE-1) and incubated for 24 h before treatment. Cells were then incubated with the indicated concentrations of various PUFAs (LA: 50, 150 μM;GLA:25, 50, 75 μM;, AA:50, 100 μM, ALA:50, 150 μM, EPA:50, 100 μM and DHA:50, 100 μM) for 48 h, at the end of which the culture supernatant was collected for the estimation of IL-6 and TNF-α content using respective ELISA kits according to the manufacturer’s instructions (Boster, China).

### Determination of free fatty acids by gas chromatography (GC)

Both PC-3 and RWPE-1 cells were inoculated in 50 ml cell culture flasks in a volume of 6 ml at a density of 1 × 10^5^ cells/ml respectively and cultured for 24 h. Then the cells were treated with selected concentrations of LA: 50, 150 μM; GLA: 25, 50, 75 μM; AA: 50, 100 μM; ALA: 50, 150 μM; EPA: 50, 100 μM; and DHA: 50, 100 μM; for 48 h. At the end of the incubation period, cells were harvested by centrifuging at 2,000 rpm for 4 min and washed twice with PBS and finally re-suspended in 500 μl PBS The cell suspension was extracted with 1 ml HCl-methanol (v:v = 5%) and the tubes were sealed and heated in incubator at 100°C for 3 h [25]. After cooling to the room temperature, 1 ml of high-purity water was added and the methyl esters were extracted with 3 ml of n-hexane in 3 portions. The n-hexane phase was collected together and transferred to another tube and diluted with the same volume of high-purity water for washing following which n-hexane phase was filtered with 0.22 μm filter membrane and transferred to another tube. The extract was evaporated under nitrogen and the residue redissolved in 100 μl n-hexane.

### Gas chromatography analysis

The methylated fatty acids in the cells were analysed using a 60 m non-polar capillary column (0.25 mm internal diameter, DB-23, Agilent, America) on an Agilent 7890A gas chromatograph fitted with a flame ionization detector (FID). 1 μL samples were injected in non-spilt mode (injector temperature: 250°C). Column temperature was held at 130°C for 1 min, and increased at a rate of 6.5°C/min to 170°C, then increased at a rate of 2.75°C/min to 225°C which was maintained for 10 min. Fatty acids were quantified by an external standard method using the animal fatty acid methyl ester mix (FAME Mix, Supelco).

### Detection of free radicals by Electron Spin Resonance (ESR)

#### The Preparation of Spin Trapping Agent PBN

The PBN needed was weighed accurately and dissolved in sterile PBS in warm bath. Then it was stirred for 30 min until dissolved completely to become PBN saturated solution with the concentration of 200 mmol/l, and saturated solution was diluted with the same volume of RPMI 1640 when used. PBN stock solutions were filtered with 0.22 μm filter membrane.

#### Trapping radicals from cells and preparation of samples for Electron Spin Resonance (ESR)

Cells were planted in 12-well plates in a volume of 2 ml at a density of 1 × 10^5^ cells/ml (PC-3 and RWPE-1) and incubated for 24 h before the treatment. Then both PC-3 and RWPE-1 cells were exposed to selected concentrations of LA (50, 150 μM); GLA (25, 50, 75 μM); AA (50, 100 μM); ALA (50, 150 μM); EPA (50, 100 μM); and DHA (50, 100 μM) for 48 h respectively. At the end of 48 hours of treatment, the medium was removed and 0.5 ml RPMI 1640 medium without fetal bovine serum was added to each well, together with 0.5 ml PBN at the final concentration of100 mmol/l in each group. The cells were incubated in 37°C for 45 min, and then scraped with sterile cell scrapers. Cell suspensions were collected into 1.5 ml centrifuge tubes and the samples were detected using ESR Spectrometer (Bruker, A300) as soon as possible.

Conditions used for detection: X waveband, Center Magnetic Field: 3385G, Sweep Length: 400G, Modulation Amplitude: 3.2G, Microwave Power: 20 mW, at room temperature.

### Statistical analysis

All results obtained were expressed in mean ± SD. Statistical analysis was performed by analysis of variance or by paired t-test when just two values were compared, using SPSS software version 16.0. Each experiment was carried out in triplicate and repeated twice. Group differences were shown as * meaning p < 0.05, ** meaning p < 0.01, *** meaning p < 0.001.

## Results

### Cell proliferation and viability

**Figure 1:**
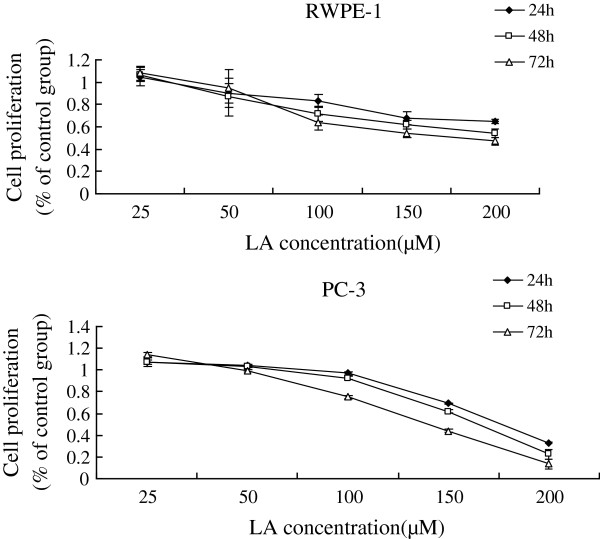
Effect of different doses of LA on the growth of RWPE-1 and PC-3 cells in vitro at 24, 48 and 72 hours.

**Figure 2:**
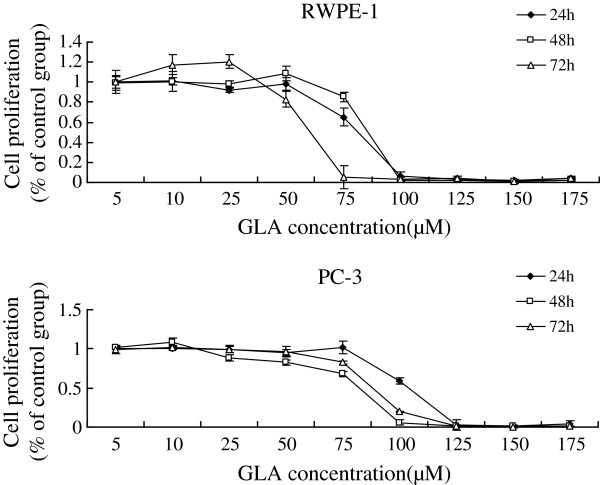
Effect of different doses of GLA on the growth of RWPE-1 and PC-3 cells in vitro at 24, 48 and 72 hours.

**Figure 3:**
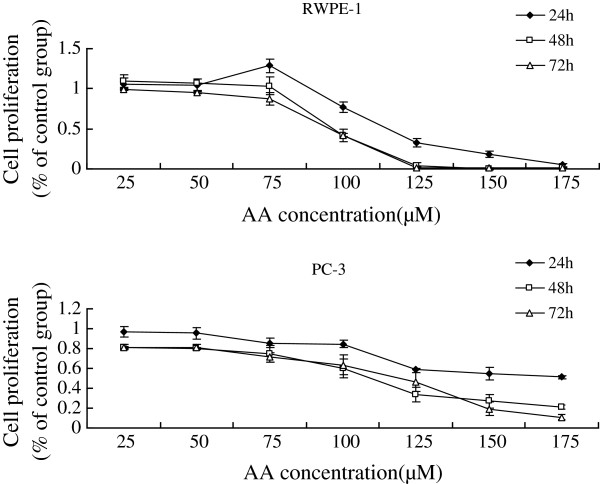
Effect of different doses of AA on the growth of RWPE-1 and PC-3 cells in vitro at 24, 48 and 72 hours.

**Figure 4:**
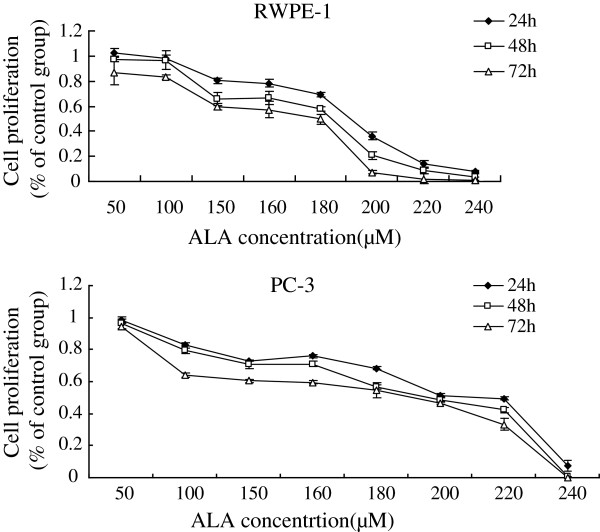
Effect of different doses of ALA on the growth of RWPE-1 and PC-3 cells in vitro at 24, 48 and 72 hours.

**Figure 5:**
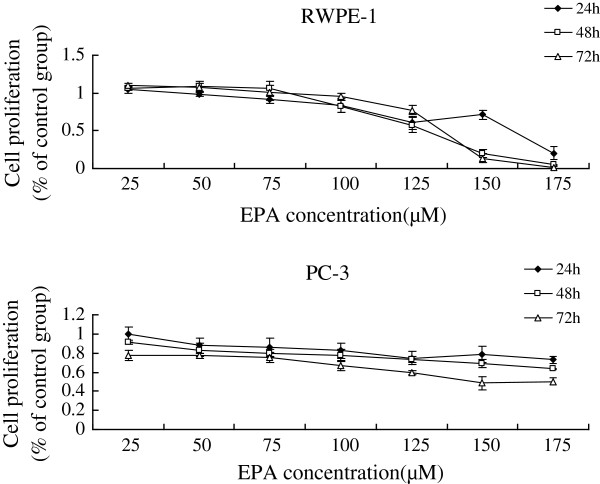
Effect of different doses of EPA on the growth of RWPE-1 and PC-3 cells in vitro at 24, 48 and 72 hours.

**Figure 6:**
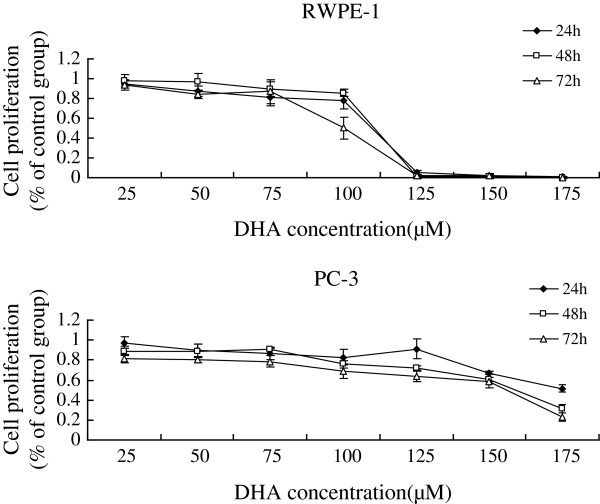
Effect of different doses of DHA on the growth of RWPE-1 and PC-3 cells in vitro at 24, 48 and 72 hours.

Supplementation of various fatty acids AA (25, 50, 75, 100, 125, 150, 175 μM), ALA (50, 100, 150, 160, 180, 200, 220, 240 μM), EPA (25, 50, 75, 100, 125, 150, 175 μM), DHA (25, 50, 75, 100, 125, 150, 175 μM) to RWPE-1 and PC-3 cells resulted in a significant decrease in cell viability when compared with the control group. Both RWPE-1 and PC-3 cells showed no significant decrease in cell proliferation at lower concentrations of both LA (below 50 μM) and GLA (below 50 μM), while the growth of RWPE-1 cells were suppressed notably at higher concentrations of LA (100,150,200 μM) and GLA (75,100,125,150,175 μM), and the proliferation of PC-3 cells were suppressed at LA (150,200 μM) and GLA (100,125,150,175 μM) (Figures 1, 2, 3, 4, 5 and 6) which suggested that RWPE-1 cells are more sensitive to the cytotoxic action of fatty acids compared to PC-3 cells.

### Fatty acid composition of RWPE1 and PC-3 cells

**Figure 7:**
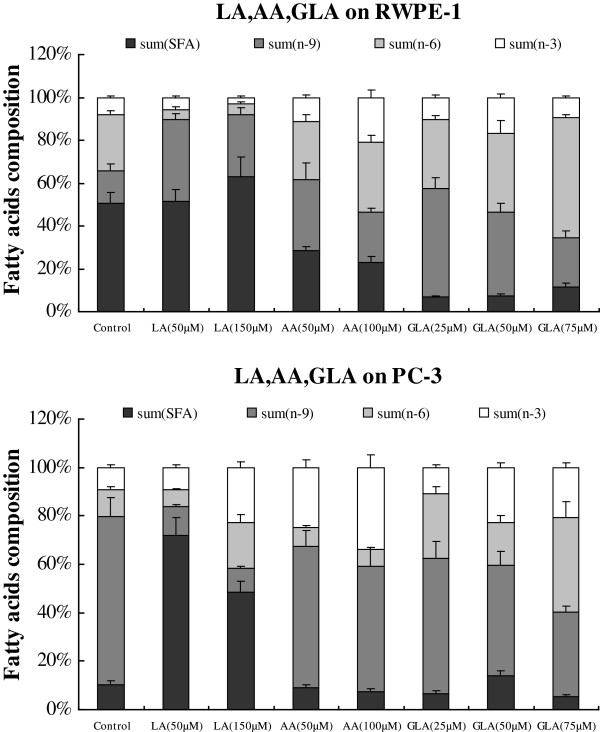
Changes in the fatty acid content of RWPE-1 and PC-3 cells that were supplemented for 48 hours with LA, GLA and AA.

**Figure 8:**
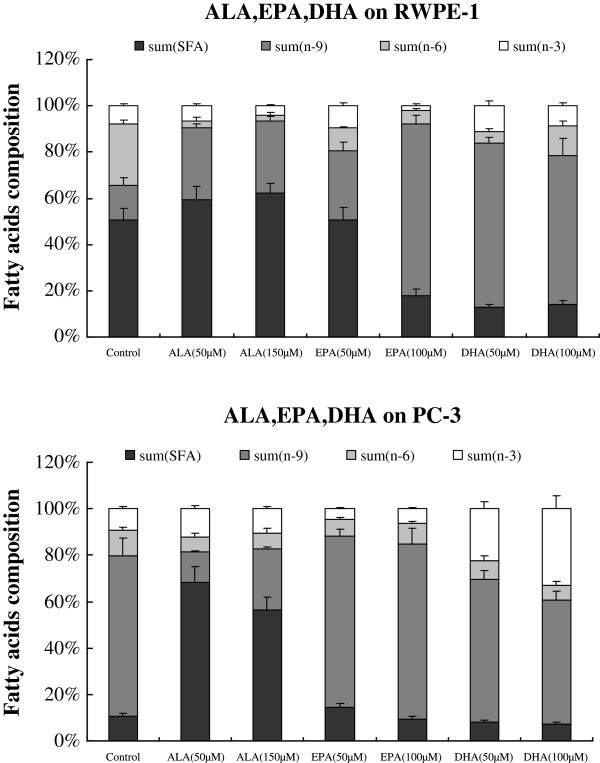
**Changes in the fatty acid content of RWPE-1 and PC-3 cells that were supplemented for 48 hours with ALA, EPA and DHA.** Results shown in Figures 7 and 8 are the mean ± SD from 3 separate experiments and each experiment in triplicate.

The fatty acid profiles of RWPE-1 and PC-3 cells were significantly different as shown in Tables 1 and 2 and Figures 7, 8. RWPE-1 cells have significantly higher amounts of LA, ALA, EPA, and DHA compared to PC-3 cells (see Tables 1 and 2 and 3).

**Table 1:** Fatty acid analysis of RWPE-1 cells that were supplemented with various fatty acids at the end of 48 hours of supplementation of various fatty acids

**Fatty acids in RWPE-1**	**Composition percentage (%)**						
	**Linolelaidic acid**	**Linoleic acid**	**γ-linolenic acid**	**Arachidonic acid**	**α-linolenic acid**	**EPA**	**DHA**
Control	2.15 ± 0.50	0.38 ± 0.09	0.76 ± 0.13	0.92 ± 0.14	0.47 ± 0.09	0.86 ± 0.13	2.58 ± 0.49
LA(50 μM)	0.12 ± 0.05***	1.59 ± 0.13**	0.49 ± 0.08*	1.76 ± 0.39**	0.19 ± 0.02*	1.73 ± 0.41*	2.60 ± 0.60
LA(150 μM)	0.18 ± 0.08***	0.52 ± 0.18*	2.62 ± 0.64**	0.70 ± 0.12	0.33 ± 0.09	0.78 ± 0.14	0.69 ± 0.06**
GLA(25 μM)	1.64 ± 0.03*	0.19 ± 0.03*	1.44 ± 0.09*	21.95 ± 2.99***	3.48 ± 0.57**	N.D	0.43 ± 0.14**
GLA(50 μM)	1.13 ± 0.02**	0.65 ± 0.06*	1.01 ± 0.13*	27.21 ± 0.02***	8.45 ± 0.19***	N.D	0.65 ± 0.01**
GLA(75 μM)	N.D	0.99 ± 0.17*	12.34 ± 0.55***	19.63 ± 1.47***	10.59 ± 1.42***	N.D	0.88 ± 0.07**
AA(50 μM)	19.58 ± 0.83***	7.3 ± 0.19**	4.57 ± 1.45**	7.01 ± 0.74**	0.69 ± 0.27*	5.52 ± 0.76***	6.63 ± 0.17**
AA(100 μM)	21.22 ± 0.29***	7.19 ± 0.18***	4.04 ± 1.10**	10.28 ± 0.43***	1.60 ± 0.22**	10.63 ± 1.27***	9.30 ± 1.68**
ALA(50 μM)	0.56 ± 0.08***	0.39 ± 0.07	0.97 ± 020	0.49 ± 0.05*	0.62 ± 0.13*	2.04 ± 0.41*	2.60 ± 0.64
ALA(150 μM)	0.17 ± 0.07***	0.53 ± 0.08	0.77 ± 0.23	0.85 ± 0.13	0.86 ± 0.10*	1.92 ± 0.60**	0.43 ± 0.05**
EPA(50 μM)	0.92 ± 0.02**	4.67 ± 0.59***	0.83 ± 0.02	N.D	2.61 ± 0.84**	1.14 ± 0.25	6.27 ± 0.34***
EPA(100 μM)	1.24 ± 0.32**	1.83 ± 0.34**	1.65 ± 0.34*	1.07 ± 0.04	0.53 ± 0.17	4.42 ± 0.43***	7.22 ± 0.44***
DHA(50 μM)	1.14 ± 0.41**	1.41 ± 0.07**	0.93 ± 0.32	2.94 ± 0.59**	N.D	N.D	14.95 ± 0.63***
DHA 100 μM	1.54 ± 0.09*	1.38 ± 0.32**	2.41 ± 0.64**	2.29 ± 0.00**	0.44 ± 0.06	N.D	15.35 ± 0.08***

*P < 0.05, **P < 0.01, ***P < 0.001 compared to control.

**Table 2:** Fatty acid analysis of PC-3 cells that were supplemented with various fatty acids at the end of 48 hours of supplementation of various fatty acids

**Fatty acids in PC-3**	**Composition percentage (%)**						
	**Linolelaidic acid**	**Linoleic acid**	**γ-linolenic acid**	**Arachidonic acid**	**α-linolenic acid**	**EPA**	**DHA**
Control	2.25 ± 0.01	0.14 ± 0.01	0.88 ± 0.03	1.22 ± 0.03	0.12 ± 0..01	N.D	0.71 ± 0.03
LA(50 μM)	0.05 ± 0.01***	0.80 ± 0.10**	0.40 ± 0.08*	1.25 ± 0.19	0.09 ± 0.02	0.89 ± 0.11**	0.58 ± 0.06
LA(150 μM)	6.05 ± 0.08**	1.92 ± 0.38***	0.42 ± 0.14*	0.88 ± 0.12*	5.58 ± 0.39***	2.48 ± 0.34***	3.02 ± 0.26**
GLA(25 μM)	1.62 ± 0.18*	0.19 ± 0.08	0.97 ± 0.08	15.29 ± 2.73***	1.83 ± 0.37**	N.D	0.50 ± 0.12
GLA(50 μM)	1.56 ± 0.10*	0.20 ± 0.02	1.08 ± 0.28	22.24 ± 0.31***	4.87 ± 0.32***	N.D	0.53 ± 0.01
GLA(75 μM)	1.11 ± 0.05**	0.16 ± 0.06	0.76 ± 0.00	29.28 ± 0.52***	10.98 ± 0.45***	N.D	0.35 ± 0.01*
AA(50 μM)	1.94 ± 0.04	0.08 ± 0.01**	0.09 ± 0.01***	14.28 ± 0.44***	0.08 ± 0.01	0.37 ± 0.01*	0.69 ± 0.09
AA(100 μM)	1.88 ± 0.04	0.08 ± 0.01**	0.08 ± 0.01***	22.20 ± 0.63***	0.07 ± 0.01	0.46 ± 0.01*	0.62 ± 0.09
ALA(50 μM)	0.60 ± 0.08***	0.45 ± 0.08*	0.72 ± 0.24	1.49 ± 0.35*	0.51 ± 0.13*	1.98 ± 0.42**	4.83 ± 0.68**
ALA(150 μM)	0.66 ± 0.27***	1.32 ± 0.20***	0.82 ± 0.33	1.13 ± 0.13	2.13 ± 0.18**	1.64 ± 0.40**	2.50 ± 0.35**
EPA(50 μM)	2.46 ± 0.65	0.26 ± 0.05*	1.35 ± 0.12*	1.17 ± 0.31	0.11 ± 0.005	2.78 ± 0.03***	9.56 ± 0.38***
EPA(100 μM)	1.79 ± 0.13*	0.24 ± 0.03*	1.16 ± 0.02*	1.37 ± 0.05*	0.14 ± 0.03	5.64 ± 1.80***	14.11 ± 2.57***
DHA(50 μM)	1.98 ± 0.10*	0.24 ± 0.02*	1.08 ± 0.03*	1.30 ± 0.02	N.D	N.D	11.17 ± 1.08***
DHA 100 μM	1.88 ± 0.04*	0.21 ± 0.02*	0.99 ± 0.09	1.32 ± 0.07	0.11 ± 0.02	0.11 ± 0.01*	19.08 ± 0.06***

*P < 0.05, **P < 0.01, ***P < 0.001 compared to control.

**Table 3:** Fatty acid content and fold increase in LA, GLA, AA, ALA, EPA and DHA in RWPE-1 and PC-3 cells supplemented with these fatty acids as compared to control

**Fatty acid supplemented**	**RWPE-1 cells**	**Fold increase compared to control**	**PC-3 cells**	**Fold increase compared to control**
Control - LA	0.38 ± 0.09	-	0.14 ± 0.01	-
LA 50 μM	1.59 ± 0.13	4.18	0.80 ± 0.10	5.71
LA 150 μM	0.52 ± 0.18	1.37	1.92 ± 0.38	13.71
Control - GLA	0.76 ± 0.13	-	0.88 ± 0.03	-
GLA 25 μM	1.44 ± 0.09	1.89	0.97 ± 0.08	1.10
GLA 50 μM	1.01 ± 0.13	1.33	1.08 ± 0.28	1.23
GLA 75 μM	12.34 ± 0.55	17.63	0.76 ± 0.01	< 1.0
Control- AA	0.92 ± 0.14	-	1.22 ± 0.03	-
AA 50 μM	7.01 ± 0.74	7.62	14.28 ± 0.44	11.70
AA 100 μM	10.28 ± 0.43	11.17	22.20 ± 0.63	18.20
Control- ALA	0.47 ± 0.09	-	0.12 ±0.01	-
ALA 50 μM	0.62 ± 0.13	1.32	0.51 ± 0.13	4.25
ALA 150 μM	0.86 ± 0.10	1.83	2.13 ± 0.18	17.75
Control- EPA	0.86 ± 0.13	-	N.D.	-
EPA 50 μM	1.14 ± 0.25	1.33	2.78 ± 0.03	>2.78
EPA 100 μM	4.42 ± 0.43	3.88	5.64 ± 1.80	>5.64
Control-DHA	2.58 ± 0.49	-	0.71 ± 0.03	-
DHA 50 μM	14.95 ± 0.63	5.79	11.17 ± 1.08	15.73
DHA 100 μM	15.35 ± 0.08	5.95	19.08 ±0.06	26.87

More than 50% of total fatty acids in RWPE-1 and PC-3 cells in the control groups and treated with LA, AA, ALA, EPA and DHA were saturated and monounsaturated fatty acids, except for RWPE-1 and PC-3 cells that were supplemented with 75 μM GLA, which contained higher content of n-6 PUFAs (56.41% in RWPE-1, 38.72% in PC-3 cells) (Figures 7 and 8). On the other hand, n-3 PUFAs content in both RWPE-1and PC-3 cells that were treated with 100 μM AA was ~ 20.72% and 33.73% respectively In contrast, the content of n-3 PUFAs in RWPE-1 cells incubated with 150 μM LA were at the lowest level (2.6%), and RWPE-1 cells treated with ALA (50, 150 μM) had the lowest contents (approx. 2.79% and 2.80%, respectively) of n-6 PUFAs.

RWPE-1 cells supplemented with n-6 PUFAs (50 μM AA and 100 μM AA; 25 μM GLA, 50 μM GLA and 75 μM GLA) resulted in 0.48, 6.31, 5.21, 10.03, 29.85% increases for the sum of n-6 PUFAs and 3.54, 12.90, 2.74, 9.00, 1.52% increases for the sum of n-3 PUFAs compared to control. However, supplementation with LA (50 and 150 μM) resulted in 21.82, 21.72% increase in the total amount of n-6 PUFAs and 1.50, 5.17% decrease in n-3 PUFAs compared to control. In contrast, supplementation with n-3 PUFAs (50 μM ALA and 150 μM ALA; 50 μM EPA and 100 μM EPA; 50 μM DHA and 100 μM DHA) to RWPE-1 cells resulted in 23.76, 23.75, 16.87, 20.36, 21.66 13.52% decrease in the total amount of n-6 PUFAs in comparison with control. In contrast, supplementation of 50 μM ALA, 150 μM ALA, and 100 μM EPA resulted in 1.20, 3.68, 6.01% decrease, while supplementation with 50 μM EPA, 50 μM DHA, 100 μM DHA resulted in 1.87, 3.47, 0.85% increase in n-3 PUFAs above control (see Figures 7 and 8; Tables 1, 2 and 3).

On the other hand, PC-3 cells that were supplemented with n-6 PUFAs (50 μM LA; 50 μM AA; and 100 μM AA) resulted in a marginal decrease of ~ 0.38, 3.69, 4.08% in the total amount of n-6 PUFAs and 3.99, 15.70, 24.49% increase in n-3 PUFAs in comparison to control. Supplementation with 25 μM GLA, 50 μM GLA and 75 μM GLA resulted in an increase in the total content of n-6 PUFAs by 15.32, 6.60, 27.54% respectively and n-3 PUFAs by 1.71, 13.30, and 11.64% respectively above the control values. In contrast, PC-3 cells supplemented with LA (50 μM) resulted in 7.72 and 4.68% decrease in the total amount of n-6 and n-3 PUFAs respectively compared to control groups. Supplementation of n-3 PUFAs (50 μM ALA and 150 μM ALA; 50 μM EPA and 100 μM EPA) decreased the percentages of the sum of n-6 PUFAs by 6.76, 6.44, 3.87, 2.46% and the sum of n-3 PUFAs by a very small amount (0.52, 1.67, 4.82, 2.87% respectively), while supplementation with DHA (50 and 100 μM) decreased the total amount of n-6 PUFAs by 3.20, 4.56% and increased the levels of n-3 PUFAs by 13.08, 23.66% respectively (see Figures 7 and 8; Tables 1, 2 and 3).

Addition of LA, GLA, AA, ALA, EPA and DHA to RWPE-1 and PC-3 cells resulted in significant changes in their content of the respective fatty acids as shown in Tables 1, 2 and 3. For instance, RWPE-1 cells, when supplemented with 150 μM LA, 75 μM GLA and 150 μM AA resulted in an increase in its LA, GLA and AA content in the cells by 1.37, 17.63 and 11.17-fold respectively. Similarly, supplementation with 150 μM of ALA, 100 μM of EPA and 100 μM of DHA enhanced their content of ALA, EPA and DHA by 1.83, 3.88 and 5.93-fold increase in the respective fatty acids’ supplemented RWPE 1 cells (Table 3). PC-3 cells supplemented with LA (150 μM), AA (100 μM), ALA (150 μM), EPA (100 μM) and DHA (100 μM) also showed 13.71, 18.2, 17.75, 5.64 and 26.87-fold increase respectively in the content of these fatty acids (Table 3). Surprisingly, supplementation of GLA75 μM did not produce any significant increase in the GLA content of PC-3 cells but showed a significant increase in its AA content suggesting that possibly, GLA is converted to its long-chain metabolite AA (Table 2).

### Changes in IL-6, TNF-α, LXA_4_ and free radical generation

**Figure 9:**
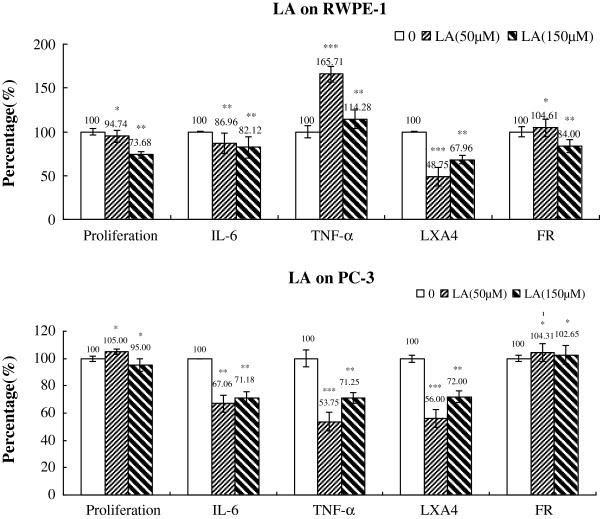
**Effect of LA on the proliferation, cytokines, LXA4 and free radical generation by RWPE-1 and PC-3 cells in vitro.** Data are presented as means ± SEM. *P < 0.05, **P < 0.01, ***P < 0.001; tTest.

**Figure 10:**
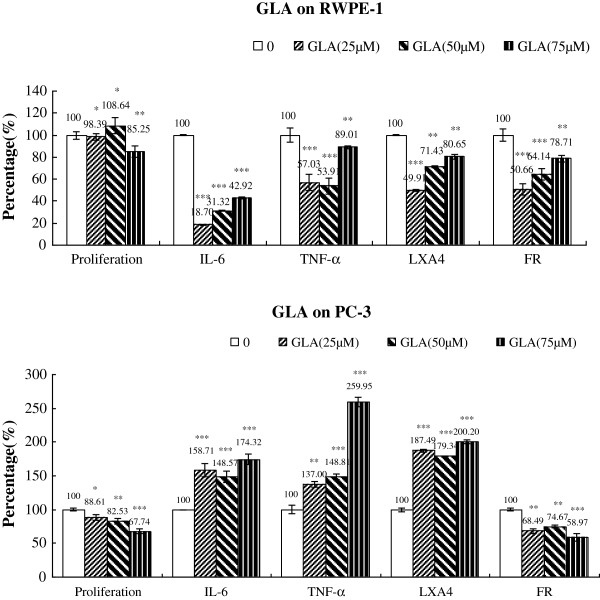
**Effect of GLA on the proliferation, cytokines, LXA4 and free radical generation by RWPE-1 and PC-3 cells in vitro.** Data are presented as means ± SEM. *P < 0.05, **P <0.01; ***P < 0.001; t Test.

**Figure 11:**
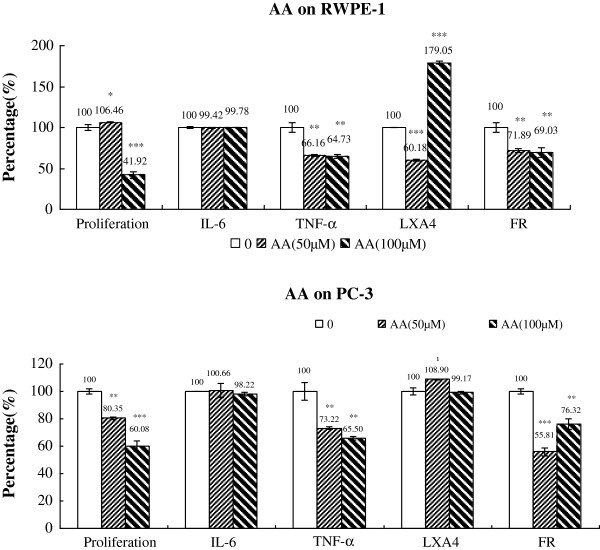
**Effect of AA on the proliferation, cytokines, LXA4 and free radical generation by RWPE-1 and PC-3 cells in vitro.** Data are presented as means ± SEM. *P < 0.05, **P <0.01; ***P < 0.001; t Test.

**Figure 12:**
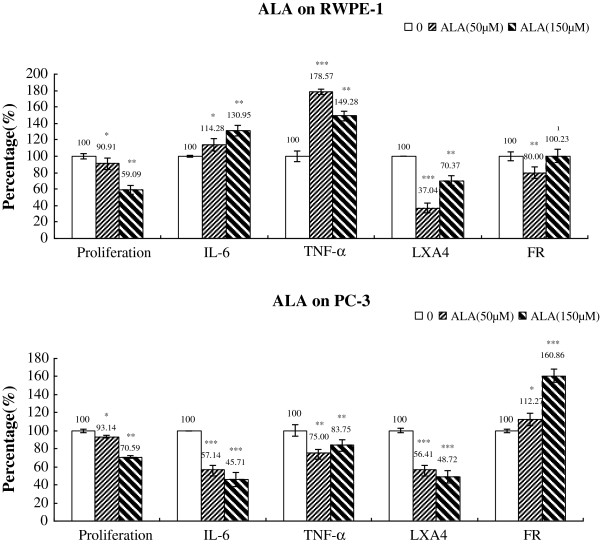
**Effect of ALA on the proliferation, cytokines, LXA4 and free radical generation by RWPE-1 and PC-3 cells in vitro.** Data are presented as means ± SEM. *P < 0.05, **P <0.01; ***P < 0.001; t Test.

**Figure 13:**
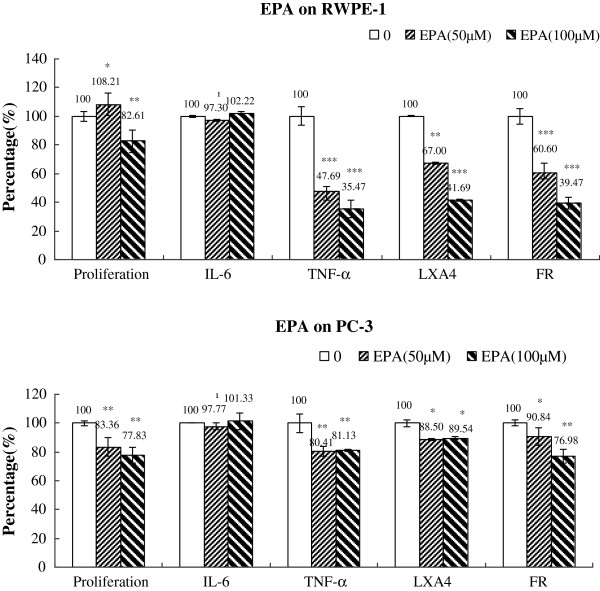
**Effect of EPA on the proliferation, cytokines, LXA4 and free radical generation by RWPE-1 and PC-3 cells in vitro.** Data are presented as means ± SEM. *P < 0.05, **P <0.01; ***P < 0.001; t Test.

**Figure 14:**
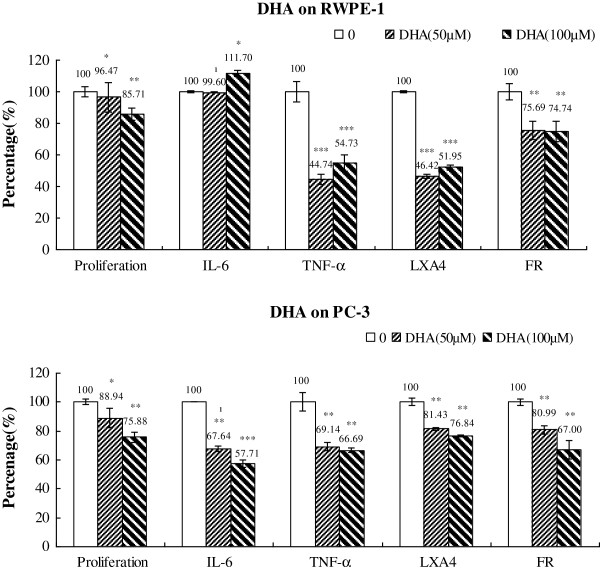
**Effect of DHA on the proliferation, cytokines, LXA4 and free radical generation by RWPE-1 and PC-3 cells in vitro.** Data are presented as means ± SEM. *P < 0.05, **P <0.01; ***P < 0.001; t Test.

IL-6, TNF-α and LXA_4_ were detectable in the culture supernatant of both RWPE-1 and PC-3 cells with and without supplementation with LA (50, 150 μM), GLA (25, 50, 75 μM), AA (50, 100 μM), ALA (50, 150 μM), EPA (50, 100 μM) and DHA (50, 100 μM) for 48 h (see Figures 9, 10, 11, 12, 13 and 14; and Tables 4, 5). Significant differences in the amount of IL-6, TNF-α and LXA_4_ released by both RWPE-1 and PC-3 cells in the presence of LA, GLA, AA, ALA, EPA and DHA was noted in comparison to control. It is interesting to find that both RWPE-1 and PC-3 cells when supplemented with LA, GLA, AA, ALA, EPA and DHA resulted in the production of widely different levels of free radicals detected by ESR.

**Table 4:** **Changes seen in the survival, FR generated, and LXA**_**4**_**, TNF-α and IL-6 secreted by RWPE-1 cells when supplemented with different doses of fatty acids for 48 hours**

**Treatment**	**RWPE-1% survival**	**FR generated % of control**	**LXA**_**4 **_**levels % of control**	**IL-6% of control**	**TNF-α % of control**
Control	100 ± 3.49	100 ± 5.40	100 ± 0.43	100 ± 0.74	100 ± 6.43
LA 50 μM	94.74 ± 7.01*	104.61 ± 10.00*	48.75 ± 10.46***	86.96 ± 11.94**	165.71 ± 8.60***
LA 150 μM	73.68 ± 3.58**	84.00 ± 7.38**	67.96 ± 4.53**	82.12 ± 11.94**	114.28 ± 10.77**
GLA 25 μM	98.39 ± 2.94*	50.66 ± 4.80***	49.91 ± 0.70***	18.70 ± 0.42***	57.03 ± 7.18***
GLA 50 μM	108.64 ± 7.30*	64.14 ± 5.10***	71.43 ± 0.79**	31.32 ± 0.51***	53.91 ± 7.47***
GLA 75 μM	85.25 ± 4.95**	78.71 ± 2.77**	80.65 ± 2.17**	42.92 ± 1.17***	89.01 ± 0.97**
AA 50 μM	106.46 ± 0.69*	71.89 ± 2.17**	60.18 ± 0.79***	99.42 ± 0.30	66.16 ± 0.94**
AA 100 μM	41.92 ± 3.93***	69.03 ± 5.77**	179.05 ± 1.95***	99.78 ± 0.23	64.73 ± 1.83**
ALA 50 μM	90.91 ± 7.35*	80.00 ± 7.34**	37.04 ± 6.00***	114.28 ± 7.49*	178.57 ± 2.85***
ALA150μM	59.09 ± 5.44**	100.23 ± 8.08	70.37 ± 6.00**	130.95 ± 6.43**	149.28 ± 5.77**
EPA 50 μM	108.2 ± 7.74*	60.60 ± 6.50***	67.00 ± 1.15**	97.30 ± 0.57	47.69 ± 3.03***
EPA 100 μM	82.61 ± 7.85**	39.47 ± 4.32***	41.69 ± 0.64***	102.22 ± 0.93	35.47 ± 6.16***
DHA 50 μM	96.47 ± 9.31*	75.69 ± 5.70**	46.42 ± 1.06***	99.60 ± 0.44	44.74 ± 3.31***
DHA100μM	85.71 ± 3.97**	74.74 ± 6.44**	51.95 ± 1.69***	111.70 ± 2.09*	54.73 ± 5.25***

*P < 0.05, **P < 0.01, ***P < 0.001 compared to control.

**Table 5:** **Changes seen in the survival, FR generated, and LXA**_**4**_**, TNF-α and IL-6 secreted by PC-3 cells when supplemented with different doses of fatty acids for 48 hours**

**Treatment**	**PC-3% survival**	**FR generated% of control**	**LXA4 levels% of control**	**IL-6% of control**	**TNF-α% of control**
Control	100 ± 1.86	100 ± 2.09	100 ± 2.60	100 ± 0.07	100 ± 6.34
LA 50 μM	105.00 ± 1.81*	104.31 ± 6.33*	56.00 ± 6.61***	67.06 ± 6.11**	53.75 ± 6.67***
LA 150 μM	95.00 ± 4.62*	102.65 ± 6.77*	72.00 ± 4.00**	71.18 ± 4.58**	71.25 ± 3.97**
GLA 25 μM	88.61 ± 4.39*	68.49 ± 3.35**	187.49 ± 2.11***	158.71 ± 9.66***	137.00 ± 3.94**
GLA 50 μM	82.53 ± 3.72**	74.67 ± 2.34**	179.34 ± 0.79***	148.57 ± 8.14***	148.81 ± 4.64***
GLA 75 μM	67.74 ± 3.81***	58.97 ± 5.11***	200.20 ± 2.94***	174.32 ± 7.32***	259.95 ± 6.95***
AA 50 μM	80.35 ± 1.21**	55.81 ± 2.99***	108.90 ± 0.39*	100.66 ± 5.24	73.22 ± 0.91**
AA 100 μM	60.08 ± 4.04***	76.32 ± 3.80**	99.17 ± 1.07	98.22 ± 1.32	65.50 ± 1.28**
ALA 50 μM	93.14 ± 1.66*	112.27 ± 6.82*	56.41 ± 5.44***	57.14 ± 4.58***	75.00 ± 3.90**
ALA150μM	70.59 ± 1.51**	160.86 ± 7.10***	48.72 ± 6.92***	45.71 ± 7.70***	83.75 ± 6.54**
EPA 50 μM	83.36 ± 6.65**	90.84 ± 6.30*	88.50 ± 0.75*	97.77 ± 2.13	80.41 ± 3.29**
EPA 100 μM	77.83 ± 5.55**	76.98 ± 4.97**	89.54 ± 1.22*	101.33 ± 5.59	81.13 ± 1.00**
DHA 50 μM	88.94 ± 6.91*	80.99 ± 2.88**	81.43 ± 1.09**	67.64 ± 1.95**	69.14 ± 2.85**
DHA100μM	75.88 ± 3.55**	67.00 ± 6.24**	76.84 ± 0.69**	57.71 ± 2.78***	66.69 ± 1.60**

*P < 0.05, **P < 0.01, ***P < 0.001 compared to control.

Supplementation with LA (50,150 μM) for 48 h, significantly lowered secretion of IL-6, TNF-α and LXA_4_ by PC-3 cells when compared with control group (Figure 9 and Table 5). Production of free radicals changed little in contrast with PC-3 cells in other treatments (GLA, AA, ALA, EPA and DHA). Similarly, LA (50 and 150 μM) decreased the secretion of IL-6 and LXA_4_ but an increase in TNF-α was noted by RWPE-1 cells (Figure 9 and Table 4).

On the other hand, treatment with GLA resulted in a significant reduction in the secretion of IL-6, TNF-α, LXA_4_ and free radicals by RWPE-1 cells (Figure 10), whereas their (IL-6, TNF-α, LXA_4_) secretion was increased in PC-3 cells in a graded fashion with increasing doses compared with control (Figure 10 and Table 5), expect for free radicals that were decreased.

Supplementation of RWPE-1 and PC-3 cells with various doses of AA showed a general inhibition of secretion of TNF-α and free radical generation; while IL-6 secretion changed little in both RWPE-1 and PC-3 cells (Figure 11 and Tables 4, 5). It is interesting to note that AA at 50 μM of AA decreased LXA_4_ production by RWPE-1 cells while 100 μM of AA enhanced LXA_4_ secretion. In contrast, PC-3 cells showed almost the same amount of LXA_4_ secretion in the presence of 50 and 100 μM of AA.

The data in Figure 12 showed that ALA has produced a gradual increase in the secretion of IL-6, TNF-α and decrease in LXA_4_ by RWPE-1 cells and rather an opposing action on the secretion of these molecules by PC-3 cells (a decrease in IL-6, TNF-α, LXA_4_, and an increase in free radical generation). In contrast to this, free radical generation by RWPE-1 cells was decreased by 50 μM ALA but was maintained at the same level as that of the control 150 μM ALA.

Supplementation of EPA to RWPE1 cells for 48 h induced a decrease in the secretion of TNF-α, LXA_4_ and generation of free radicals with no significant change in IL-6. On the other hand, EPA induced little or no significant alterations in the secretion of IL-6, TNF-α and LXA_4_ by PC-3 cells but a slight decrease in the production of free radicals was noted at the doses tested (Figure 13 and Table 5).

When RWPE-1 and PC-3 cells were supplemented with DHA (50 μM and 100 μM), as shown in Figure 14, there was a reduction in the secretion of IL-6, TNF-α, LXA_4_ and generation of free radicals by PC-3 and RWPE 1 cells except that in RWPE-1 cells there was little change in the secretion of IL-6.

## Discussion

Epidemiological studies have suggested that dietary fat consumption is a major contributor to the risk of development and progression of prostate cancer. But the influence of n-3 and n-6 fatty acids on the risk of development of prostate cancer is controversial. Our present study aimed to delineate the effects of n-3 (ALA, EPA, DHA) and n-6 fatty acids (LA, GLA, AA) on the growth of prostate cancer PC-3 cells and its normal counterpart RWPE-1 cells and influence of these fatty acids on the generation of inflammatory cytokines, free radicals and LXA_4_, a potent anti-inflammatory lipid molecule. It is evident from the results that both n-3 (ALA, EPA, DHA) and n-6 PUFAs (LA, GLA, AA) suppress proliferation of both PC-3 and RWPE-1 cells, while these fatty acids at low concentrations had little influence on cell viability and even promoted their growth. RWPE-1 cells were more sensitive to the growth inhibitory actions of n-3 (ALA, EPA, and DHA) and n-6 fatty acids (LA, GLA, AA) in comparison to their effect on PC-3 cells, implying that RWPE-1 cells are more susceptible to the cytotoxic action of fatty acids compared to PC-3 cells.

Prostate cancer develops and progresses in response to inflammation during the cancer process. This suggests that inflammation is closely linked to prostate cancer development. Several studies showed that a pro-inflammatory environment may be set in the prostate gland tissue when the balance between pro- and anti-inflammatory PUFAs (n-6 PUFAs vs n-3 PUFAs) is tilted more towards the pro-inflammatory (n-6 PUFAs) fatty acids. N-3 PUFAs: ALA, EPA and DHA have the ability to displace AA from the cell membrane phospholipids and suppress the production of pro-inflammatory eicosanoids. But, it needs to be noted that AA not only forms precursor to pro-inflammatory prostaglandins, leukotrienes and thromboxanes but can also give rise to LXA_4_, a potent anti-inflammatory compound. Since cancer is associated with low-grade systemic and local inflammation, we evaluated whether the growth inhibitory actions shown by various fatty acids could be attributed to their ability to alter the formation of LXA_4_ both in the normal and cancer cells. The results of the present study showed that LA, ALA, EPA and DHA and LA GLA, AA, EPA and DHA decreased secretion of LXA_4_ by PC-3 cells and RWPE-1 cells respectively, while GLA enhanced and AA had very little effect on the secretion of LXA_4_ in PC-3 cells. These results suggest that, in general, most of the n-3 and n-6 fatty acids decrease LXA_4_ secretion by both normal and tumor cells with the exception of GLA and AA. These results underscore the complex nature of interaction among various PUFAs (both n-3 and n-6 PUFAs), their pro- and anti-inflammatory products (such as prostaglandins, leukotrienes and thromboxanes and lipoxins), local and systemic inflammatory process and the growth of prostate cancer cells.

IL-6 and TNF-α are pleiotropic cytokines that function as autocrine or paracrine growth factors, which are secreted by normal prostate epithelial and cancer cells. Androgen-refractory prostate cancer cells have been shown to produce detectable amounts of IL-6 [26]. N-3 and n-6 PUFAs modulate inflammation by influencing the production of IL-6 and TNF-α [27-29]. In the present study, n-6 fatty acids (LA, GLA) inhibited the production of IL-6 by RWPE-1 cells, while ALA, EPA and DHA did not suppress IL-6 secretion, while ALA enhanced IL-6 secretion (ALA > DHA > EPA). On the other hand, LA decreased but GLA increased IL-6 secretion by PC-3 cells, with no change in IL-6 secretion by AA. TNF-α secretion was increased by LA, but decreased by GLA and AA by RWPE-1 cells, whereas ALA increased but both EPA and DHA decreased the same. In PC-3 cells, LA and AA decreased TNF-α secretion whereas all the three n-3 fatty acids (ALA, EPA and DHA) decreased it by PC-3 cells. Thus, GLA inhibited and ALA enhanced the secretion of IL-6 and TNF-α by RWPE-1 cells, which showed the opposite results in PC-3 cells. However, all other fatty acids (except for LA on RWPE-1) suppressed the secretion of TNF-α at all the doses tested by both RWPE-1 and PC-3 cells , indicating that, in general, both n-3 and n-6 fatty acids inhibit the secretion of TNF-α by both normal and tumor cells *in vitro*. These results are interesting in the light of the controversy as to the role of n-6 PUFAs on tumor development since in the present study it was noted that LA and AA inhibited while GLA enhanced TNF-α secretion. These results suggest that the local concentration of n-6 PUFAs can modulate the secretion of TNF-α that, in turn, influence the local inflammatory process and consequently the process of carcinogenesis and growth of the tumor cells depending on the ratio among various n-6 PUFAs: LA, GLA and AA.

LA is the precursor for AA, which can be metabolized to pro-inflammatory and anti-inflammatory products whereas ALA is the precursor of EPA that can displace AA which may result in decreased production of pro-inflammatory eicosanoids form AA. Thus, supplementations with GLA, ALA and/or EPA modulate the inflammatory response(s). However, the results of our current study are inconsistent with what was mentioned above, which suggest that there could exist other unidentified pathways of lipid metabolism that may participate in the process of carcinogenesis and tumor cell growth. It is also likely that influence of various n-3 and n-6 lipids on the inflammatory response and process could be different in different types of cells.

It is known that supplemented fatty acids are incorporated into the cell membrane lipid pool that may influence secretion of various cytokines, free radicals and growth characteristics. Hence, we performed fatty acid analysis of both RWPE-1 and PC-3 cells that were supplemented with various fatty acids. As expected, there were significant changes in the fatty acid composition of both RWPE-1 and PC-3 cells that were supplemented with various fatty acids. It is evident from these results that supplementation of PUFAs to both RWPE-1 and PC-3 cells produced a significant increase in the respective fatty acids in both types of cells. For instance, supplementation of RWPE-1 and PC-3 cells with LA 50 μM and 150 μM produced approximately a 5-fold and 1.4 fold increase in LA content of RWPE-1 cells and a 5-fold and 8-fold increase in LA content of PC-3 cells respectively compared to respective controls. Similar significant increase in the levels of supplemented fatty acids (Viz., GLA, AA, ALA, EPA and DHA) in RWPE-1 and PC-3 cells was noted. One significant observation when the incorporation of supplemented fatty acids by RWPE-1 and PC-3 cells noted was that all the supplemented fatty acids were not incorporated to the same extent by these cells. Certain fatty acids were incorporated by RWPE-1 and PC-3 cells to a much larger extent compared to others. This is evident from the data shown in Table 2. It can be seen from the results shown in Table 2 that supplementation of fatty acids produced anywhere from 1.3 fold to 26-fold increase in the fatty acid content of the specific fatty acid supplemented.

Furthermore, supplementation ALA (both 50 and 150 μM) enhanced the content of ALA and EPA but not of DHA; EPA enhanced the content of both EPA and DHA; whereas DHA increased the content of only DHA in RWPE-1 cells significantly. On the other hand, RWPE-1 cells when were incubated with LA, a significant increase in the content of LA and GLA occurred with no change in that of AA; GLA induced a significant increase of GLA and AA; whereas supplementation of AA increased those of LA, GLA and AA. These results suggest that to certain extent, the supplemented fatty acids such as ALA, EPA and LA, and AA are metabolized to their long –chain metabolites namely ALA to EPA and DHA; EPA to DHA; LA to GLA and GLA to AA. The surprising observation that supplementation of AA to RWPE-1 cells led to an increase in LA and GLA content raises the interesting possibility that there is some amount of retroconversion of AA to GLA and LA. It is interesting to note that supplementation of AA to RWPE-1 cells led to an increase in their content of ALA, EPA and DHA (especially when they were supplemented with 100 μM) from 0.47 ± 0.09 in control to 1.60 ± 0.22 of ALA; from 0.86 ± 0.13 to 10.63 ± 1.27 of EPA and from 2.58 ± 0.49 to 9.30 ± 1.68 of DHA suggesting that a close interaction exists between the metabolism of n-3 and n-6 fatty acids, though such dramatic changes in the content of n-6 fatty acids was not noted when RWPE-1 cells were supplemented with n-3 fatty acids ALA, EPA and DHA (see Table 1).

In a similar fashion, PC-3 cells supplemented with LA (especially with 150 μM) showed an increase in ALA, EPA and DHA with a concomitant significance increase in LA; while supplementation with GLA and AA (at all the doses tested) produced a significant increase in their content of AA (Table 2), suggesting that GLA is being elongated and desaturated to AA. GLA supplementation enhanced the ALA content of PC-3 cells with little or no change in EPA and DHA and if at all there is any change a decrease in their DHA content was noted, suggesting that GLA is able to block the conversion of ALA to its long-chain metabolites EPA and DHA that could have enhanced its (PC-3 cells) ALA content (see Table 2). Supplementation of PC-3 cells with ALA, EPA and DHA enhanced their content of EPA and DHA and of only DHA in DHA-supplemented cells suggesting that there is no retroconversion of DHA to EPA in the latter. Increased levels of EPA and DHA in ALA-supplemented PC-3 cells indicate that ALA is being elongated and desaturated to EPA and DHA in these cells. These results indicate that the way n-3 and n-6 fatty acids are handled by RWPE-1 and PC-3 cells are quite different.

In conclusion, our data suggest that there are significant differences in the way RWPE-1 and PC-3 cells metabolize n-6 and n-3 fatty acids, their ability to secrete inflammatory cytokines in the presence of various fatty acids and *de novo* fatty acid synthetic pathways. Though we could not identify the precise mechanism by which n-3 and n-6 fatty acids are able to bring about their cell killing effect since none of the indices studied (free radicals generated, changes in the levels of LXA_4_, IL-6 and TNF-α secreted) showed any direct correlation among tumor cell survival and the indices studied, it is likely that several mechanism(s) may be at play that include: generation of significant amounts of free radicals, formation of higher amounts of lipid peroxides in PUFA-supplemented cells, changes in the formation and secretion of anti-inflammatory cytokines and the response of cells to the growth enhancing potential of these cytokines, and the ability of these cells to secrete anti-inflammatory bioactive lipids such as lipoxin A_4_. Obviously, more in-depth studies are needed to understand the mechanism(s) involved in the cytotoxic action of n-3 and n-6 fatty acids on prostate cancer cells and prostate normal cells.

## Competing interests

The authors declare that they have no competing interests.

## Authors’ contributions

SRS and UND conceived the idea and designed the experiments. HZM and YZS performed the experiments and drafted the manuscript. JHS and FZ participated in the design of the study and performed the statistical analysis. SRS and UND performed the interpretation of the data. All authors read and approved the final manuscript.
